# The cascade of care of HIV after one year of follow‐up in a cohort of HIV‐positive adult patients in three health settings of Morrumbene in rural Mozambique

**DOI:** 10.1111/tmi.13671

**Published:** 2021-09-12

**Authors:** Paola Magro, Carlo Cerini, Aldorada da Gloria, Stelio Tembe, Francesco Castelli, Lina Rachele Tomasoni

**Affiliations:** ^1^ Division of Infectious and Tropical Diseases Department of Clinical and Experimental Sciences University of Brescia Brescia Italy; ^2^ NGO Medicus Mundi Italia ONLUS Brescia Italy; ^3^ Direção Provincial de Saúde de Inhambane Mocambique Italy; ^4^ Cattedra UNESCO “Training and Empowering Human Resources for Health Development in Resource‐limited Countries" University of Brescia Brescia Italy; ^5^ Division of Infectious and Tropical Diseases ASST Spedali Civili di Brescia Brescia Italy

**Keywords:** adherence, Africa, ART, cascade of care, HIV, mozambique

## Abstract

**Objective:** To assess the state of the retention in care of HIV patients in three health settings in Morrumbene, a rural district of Inhambane Province, Mozambique. We evaluated potential factors associated with early loss to follow‐up (LTFU), retention in care and ART adherence during the first year of follow‐up.

**Material and methods:** Retrospective, cross‐sectional, observational study. We collected data on patients diagnosed with HIV infection in 2017 in two permanent clinics and one mobile clinic. Demographic, clinical, immunological and therapeutic data were retrieved up to December 31st, 2018. Data on follow‐up were collected at 6 and 12 months for medical visits and for ART adherence and analysed for factors associated with LTFU, retention in care and adherence to ART by Stata Version 14 and univariate and stepwise multiple unconditional logistic regression models.

**Results:** In 2017, 960 patients were diagnosed with HIV infection. At 6‐month follow‐up, 49% attended the medical visit and 157 (25%) adhered to ART. After one year, 34% of patients were available for follow‐up, and only 72 patients adhered to ART. In multivariate analysis, factors associated with early LTFU were male sex (*p* = 0.036) and immediate prescription of ART (*p* = 0.064). Older age (*p* < 0.001) and being followed in the mobile clinic (*p* = 0.001) favoured retention in care. Advanced WHO status (*p* = 0.005) and being pregnant or breastfeeding showed a negative correlation with adherence to treatment (*p* = 0.068).

**Conclusions:** Only one‐third of patients were available for follow‐up after one year, and only 13% adhered to ART. Young individuals, men and pregnant/breastfeeding women seem to be particularly at risk for LTFU and non‐adherence to treatment.

## INTRODUCTION

To date, 37.7 million people live with HIV infection worldwide [1]. Sub‐Saharan Africa (SSA) bears the highest burden of HIV, especially eastern and southern Africa, which are home to 54% of people living with HIV. Here, the numbers still reflect the massive proportions of the epidemic: 20.6 million adults and children live with HIV infection, 675,000 people acquired HIV infection and 310,000 died of AIDS‐related diseases in 2020 [1]. In Mozambique, 2.2 million people live with HIV infection. HIV prevalence among the adult population aged 15–49 years is 13.2%, being higher among women (15.4%) than men (10.1%) [2]. ART coverage has been increasing in Mozambique in the last decades, reaching 54% in 2016 [3]. Rates of retention in care and adherence to ART are difficult to assess and need to take into account the wide variability of the different underlying contexts. A recent study from Lafort et al [4] in Mozambique highlighted the scarcity of data on the continuum of HIV care from peripheral health centres without electronic patient tracking systems (EPTS). Here, HIV patients in more remote contexts were more likely to be lost to follow‐up (LTFU) than those followed in bigger centres (51.8/100PY vs. 37.7/100PY). High rates of LTFU are moreover consistent with other studies from Mozambique and SSA [5, 6]. With the aim of reaching remote populations with scarce or no access to HIV health services, in 2017 the non‐governmental organisation (NGO) Medicus Mundi Italia (MMI), in collaboration with local health authorities (*Serviços Distritais de Saúde Mulher e Ação Social e Direcção Provincial de Saúde*) designed a strategy called “TARV Movel” service (TM) in the district of Morrumbene, Province of Inhambane, Southern Mozambique. Taking advantage of the long multi‐year experience of MMI on supporting the Mobile Clinics strategy (*Brigadas Móveis*), that usually offers primary healthcare services for more distant rural areas, populous communities were selected to receive the HIV outpatient package, in full compatibility with the protocols of the Mozambican Ministry of Health (MISAU). By the time of our analysis, TM served the remote locations of Chicungussa, Mucambe Feha and Bie. The package included counselling and clinical visits, ART dispensation and laboratory analysis, and was offered once a month, all fully integrated with other primary health services, such as vaccinations and general visits. With this strategy, HIV patients had the opportunity to being attended in a health centre or in the mobile clinic, with the same standard of care and specific tailored services, such as lymphocytes CD4+ count on‐site. MMI offered coordination of the activity and technical supports to all the phases of the activity. In the framework of the collaboration between the University of Brescia UNESCO Chair and MMI, we evaluated the current state of the HIV cascade of care in the TMs and in two other types of health service delivery: At the first level, central Health Center of Morrumbene City (CS); and at the second level, the peripheral Health Center of Mahangue (MAH). We then performed two analyses, with two different but interconnected aims: first, a snapshot of the HIV continuum of care in the three settings in the rural district of Morrumbene, to identify potential weaknesses and strengths of the three different strategies. Secondly, we evaluated whether there were any epidemiological, social, clinical and therapeutic factors correlated with LTFU, retention in care and the adherence to antiretroviral treatment in the overall population, to identify risk groups on which future strategies should focus.

## MATERIALS AND METHODS

### Ethical approval

The study was conducted in accordance with the guidelines of the Declaration of Helsinki and the principles of Good Clinical Practice. The study protocol was approved by the local authorities that released an ethical and formal authorisation on collecting and analysing data (Nota no. 67/001.5/DPSI/NIOI/2021). Written or oral informed consent was obtained from all patients at the time of enrolment, as part of the routinely activities of health centres of the National Health System, according to local regimentations. Data from this study were approved for publication (Nota no. 67/001.5/DPSI/NIOI/2021).

### Study design and settings

We performed a retrospective, observational study. The study took place in the rural Morrumbene district in the province of Inhambane, southern Mozambique. In 2017, Morrumbene was home to 152,517 people, 56% of whom were women [7].

We collected data on all adult patients aged ≥14 years old with a new diagnosis of HIV infection performed between the 1st of January and the 31st of December 2017 in three health facilities of the Morrumbene district:

‐ The first level Centro de Saúde de Morrumbene (CSM)
‐ The second level Centro de Saúde de Mahangue (MAH)
‐ the TARV Móvel service (TM).

For the retrospective nature of this work, the three populations hailing from the three health centres were not matched for any characteristics. As described in another study [4], the smaller type III rural health centres offer only basic care, type II health centres have a maternity department and type I centres hold an in‐patient ward. The TM service is a mobile clinic that reaches three small rural villages once a month (Chicungussa, Mocambe Feha and Bie by the time of the current study), making HIV services and ART available in these remote locations. The study was conducted in the context of the health cooperation project "CAREvolution: innovation of community health services in Inhambane Province" (AID Code 11492) [8].

### Data collection

Data were collected manually through paper and digital medical records available on Open Medical Record System (OpenMRS®). Digital records were available only for patients followed by CSM and TM. We retrieved data on age, sex, education, pregnancy, breastfeeding by the moment of HIV diagnosis, date of HIV diagnosis, date of entry to care, site and centre of diagnosis of HIV infection, CD4+ cell count and WHO status at baseline, date of ART prescription, presence at visit, death, transfer to other health centre and adherence to ART.

### Study design

We evaluated three steps of the cascade of HIV care. Regarding timing and prescription of ART, the criteria for eligibility for ART were consistent with the national guidelines [9] (Table 1). In the second half of 2017, the national criteria for ART initiation changed, and ART was made available for every patient with a diagnosis of HIV infection, independently of WHO status and/or CD4+ cell count [10].

**TABLE 1:** Criteria for ART initiation in adult patients [9]

Criteria for art iniciation		
WHO status	CD4+ available	CD4+ not available
I	Start if CD4+ ≤500 cell/mm^3^	Don't Start ART
II		
III	Start with any CD4+ cell count	Start ART
IV		
Special groups		
Every pregnant and breastfeeding woman should be started on ART independently from WHO status and CD4+ cell count		
All patients co‐infected with HBV or HTLV should start TARV independently from WHO status and CD4+ cell count		
All patients with TB co‐infection with any localization should start TARV independently from WHO status and CD4+ cell count		
All patients with a diagnosis of any invasive cancer should start TARV independently from WHO status and CD4+ cell count		
All HIV positive partners of pregnant and/or breastfeeding women should start TARV independently from WHO status and CD4+ cell count		

*Immediate initiation of ART* was defined as starting an antiretroviral regimen on the same day of the HIV diagnosis. As for retention in care, we evaluated the adherence to the scheduled follow‐up at 6 and 12 months after HIV diagnosis (Table 2). We documented whether patients were transferred to other centres or reported as dead in their medical records.

**TABLE 2:** Criteria for the definition of adherence to follow‐up

Retention in care
HIV‐positive patients are routinely programmed with a medical visit each month. We considered adherent patients who:
1. attended the visit at the 6th and the 12th month as scheduled, with a maximum delay of one month, or as agreed with the healthcare practitioner
or
2. Regularly collected the prescribed ART even if not attending medical visit.
Adherence to ART
HIV‐positive patients normally collected their prescribed ART at the pharmacy once a month. We considered adherent those patients that collected their ART each month for six consecutive months at 6 and 12 months since the prescription.
Lost to follow‐up
We considered lost to follow‐up those patients who were not present at one or both scheduled visits and who did not pick‐up their ART by the time of the observation, irrespectively of their ART prescription. Patients meeting such criteria both at 6th and at 12th months were classified as *early lost*.

Regarding adherence to ART, we evaluated rates of adherence to ART for the last step of the cascade as a surrogate for viral suppression. Conscious of the strong limitations of this choice that we will further discuss, we chose to evaluate adherence to ART because measures of the plasma viral load were not routinely available for most patients (Table 2).

Moreover, we evaluated whether there were any epidemiological, social, clinical and therapeutic factors associated with three different outcomes:

‐ Being early LTFU (Table 2), in order to evaluate which populations are more at risk to “fail” the engagement phase of the cascade of HIV care;
‐ Being retained in care, to evaluate those patients who are adherent to medical follow‐up;
‐ Being adherent to ART, to evaluate a surrogate of the viral suppression in the examined population.

### Statistical analysis

Data were stored in Excel ™ and analysed in Stata version 14. Descriptive statistics, in particular counts and proportions, mean with standard deviation (SD), median with interquartile range, were obtained as appropriate for all variables; for comparison, chi‐square test or Fisher's exact test for categorical variables, Student's *t*‐test and ANOVA test for numeric variables, were applied as appropriate. Two‐tailed tests were used. Only *p*‐values <0.05 were considered statistically significant.

The time pattern of cART prescription by site and by gender was studied using Kaplan–Meier method and by log‐rank test. In order to evaluate the factors associated with the outcomes of early loss at follow‐up, retention in care and adherence to ART, we performed a univariate logistic regression with the following variables: age, sex, pregnancy/breastfeeding, level of education, immunological and clinical status, timing and prescription of ART, being followed in one of the three health centres. For the same outcomes, a stepwise multivariable unconditional logistic regression model was applied, with a *p*‐value for entrance = 0.15 and for exit = 0.10. In the latter analysis, the site of follow‐up was retained obligatory independently from the existence of a significant correlation in the univariate analysis.

It must be said that, for many patients, medical and therapeutic records were incomplete due to incomplete collection of data at the moment of the visit or to the decay, loss or transfer of the paper medical/therapeutic record. Hence, we specify how many patients we were able to take into account in the analysis in each section.

## RESULTS

### Characteristics of the population at baseline

In 2017, 960 adult patients (≥14 years) were diagnosed with HIV infection. Females constituted 73% and 14% were pregnant. At baseline, median age was 36 years (28–75 IQR), median CD4+ T‐cell count was 309 cells/ml (155–510 cells/ml IQR). Two hundred and eighty‐three (32%) patients had a CD4+ T‐cell count <200 cells/ml. Males had a higher median age at diagnosis (40 vs. 33, *p* < 0.001), a lower median count of CD4+ T cells (273 vs. 325 cell/ml, *p* < 0.001) and a more advanced WHO status (24% vs. 11%, *p* < 0.001, had III or IV stage), whereas females tended to have a lower level of education (Table 3). The CSM population had the highest percentage of males (29%) and the highest proportion of patients diagnosed with an advanced WHO status (19% versus 4% and 5% in TM and MAH respectively). MAH had the highest proportion of pregnant women (20%), with female patients being significantly younger than in other centres (*p* = 0.02). In TM, one‐third (29%) of the patients had no educational at all, especially females (33%, data not shown). CD4+ T‐cell count at baseline was not significantly different among the three centres, although the proportion of patients with <100 CD4+ T cells/ml was larger in CSM (*p* = 0.015) (Table 3).

**TABLE 3:** Demographic, clinical and immunological characteristics of patients at baseline

	ALL	Data (n)	CSM	Data (n)	MAH	Data (n)	TM	Data (n)
Male (n, %)	259 (27%)	960	198 (28.9%)	684	45 (26.9%)	167	16 (14.7%)	109
Age (years, median, IQ)	36 (28–75)	960	36 (28–72)		32 (27–75)		38 (26–68)	
Pregnancy (n. %)	96 (13.7%)	701	66 (13.6%)	486	24 (19.7%)	122	6 (6.5%)	93
Education (n, %)								
None	166 (21.6%)	768	138 (24.2%)	571	4 (3.5%)	114	24 (28.9%)	83
Secondary or more	254 (33.1%)	768	188 (32.9%)	571	48 (42.1%)	114	18 (21.7%)	83
WHO status (n, %)								
WHO I	581 (61.8%)	940	349 (52.5%)	665	147 (88.6%)	166	85 (78%)	109
WHO II	220 (23.4%)	940	189 (28.4%)	665	13 (7.8%)	166	18 (16.5%)	109
WHO III/IV	139 (14.8%)	940	127 (19.1%)	665	6 (3.6%)	166	6 (5.5%)	109
CD4+ T cells (n cell/ml) (median, IQ)	309 (156–510)	894	308 (140–494)	654	320 (203–562	133	306 (185–555)	107
CD4+ T cells <200 cell/ml (n, %)	283 (31.7%)	894	221 (33.8%)	654	33 (24.8%)	133	29 (27.1%)	107
CD4+ T cells <350 cell/ml (n, %)	497 (55.6%)	894	367 (56.1%)	654	73 (54.9%)	133	57 (53.3%)	107
CD4+ T cells <100 cell/ml (n, %)	133 (14.9%)	894	111 (17%)	654	12 (9%)	133	10 (9.3%)	107

Abbreviations: CSM, Centro de Saude de Morrumbene; MAH, Centro de Saude de Mahangue; TM, TARV Movel service; WHO, World Health Organisation.

### Prescription and timing of ART initiation

**FIGURE 1:**
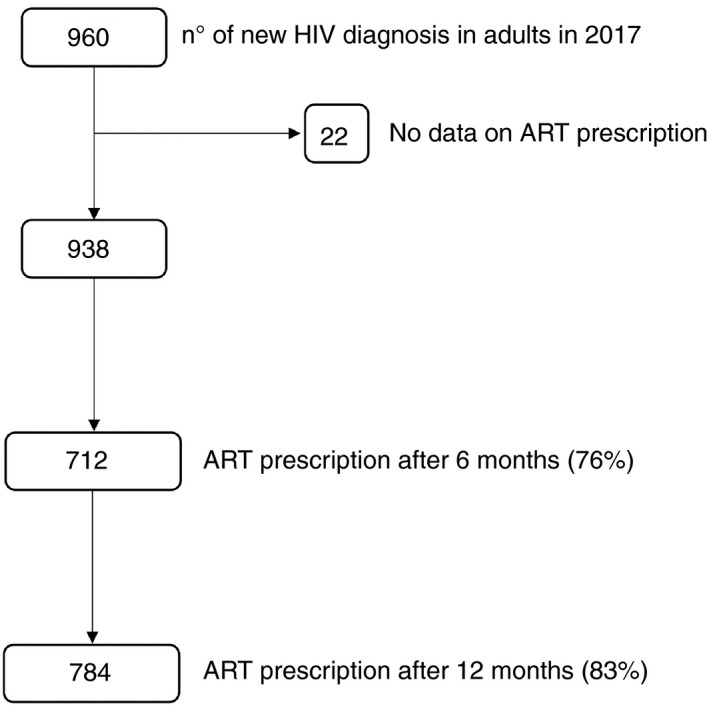
Rates of ART prescription at 6 and 12 months after HIV diagnosis

**FIGURE 2:**
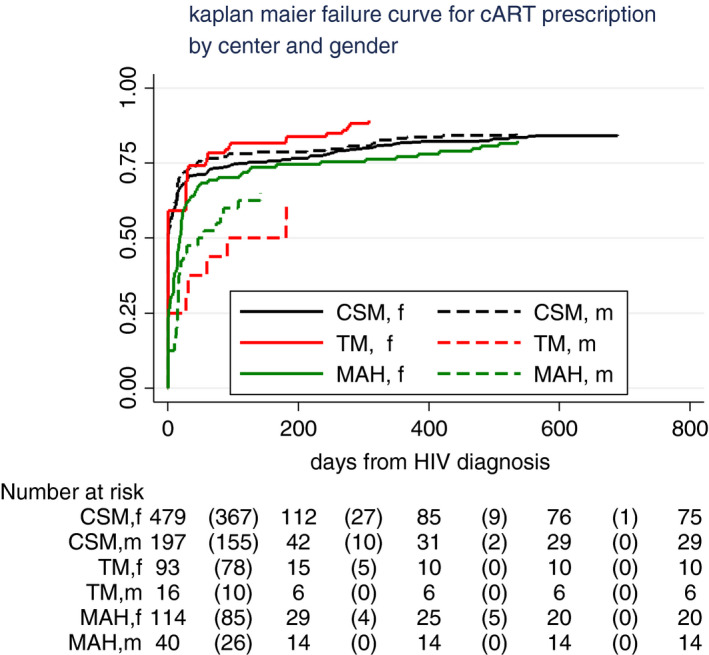
cART prescription by health centre and sex

After 6 months, 76% and by the end of 2018, 83% of the enrolled patients were prescribed ART (784/938) (Figure 1). Prescription timing differed (log‐rank test, *p* = 0.006) by health service and by sex (Figure 2): Patients who had an *immediate prescription* of ART were 52% (95%CI 48–55) at CSM, 54% (95%CI45–63) at TM and 21% (95%CI15–28) at MAH. Among females, pregnant women were more likely to be prescribed ART on the same day than other women (AOR 17, *p* < 0.001). Using stepwise logistic regression adjusted for sex, we found that patients followed in MAH (AOR 0.2, 95%CI 0.12–0.33, *p* < 0.001), elderly patients (AOR 0.9 for each 5 years of age, *p* = 0.001) and those with higher CD4+ cell count (AOR 0.86 for each 100, *p* < 0.001) were less likely to be prescribed ART on the day of HIV diagnosis. Immediate prescription of ART increased during the study period (AOR 1.7, *p* < 0.001, for the second part of 2017).

### Retention in care 6 and 12 months after HIV diagnosis

**FIGURE 3:**
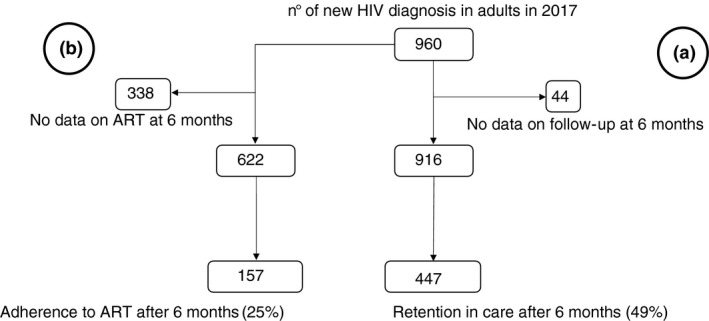
(a) Retention in care at 6 months after HIV diagnosis; (b) Adherence to ART after 6 months from ART prescription

Patients showing up at the medical visit scheduled at 6 months were 49% (447 of all eligible patients, Figures 2a and 3a), but 59% if followed in TM. During this period, 7% of TM patients were referred to other centres vs. 2% in both CSM and MAH. There was one reported death in TM, and six in CSM, accounting for 1% of the population in both centres.

When we explored how many patients were adherent at scheduled follow‐up, we counted only 297/880 (33.8%) present at both the 6‐ and 12‐month visits, with no differences among the centres. Among the 469 patients not attending the 6‐month visit, only 51 (11%) showed up at the 12‐month visit. After one year, patients registered as referred to other centres accounted for 27% of the cohort in TM, 4% in MAH and 2% in CSM (*p* < 0.001). Overall, 11 deaths (1.25%) were recorded: seven in CSM, one in MAH and three in TM, with no significant differences between the services.

### Adherence to antiretroviral treatment after 6 and 12 months from ART prescription

**FIGURE 4:**
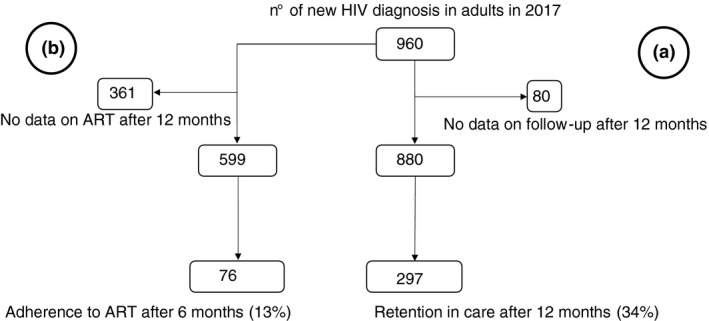
(a) Retention in care at 12 months after HIV diagnosis; (b) Adherence to ART after 12 months from ART prescription

After six months, 157 (25%) regularly collected drugs for six consecutive months, with no significant differences among the three health centres. Continuous adherence to the prescribed ART was recorded for just 76/599 patients (13%). No statistical difference was shown between CSM and TM. Conversely, patients in follow‐up in MAH were less likely to adhere to ART (Figures 3b and 4b).

### Uni‐ and multivariate analysis of factors associated with early loss, retention in care and ART adherence after one year of follow‐up

The univariate analysis for factors correlated to early loss to follow‐up (418/916) is shown in Table S2. A stepwise logistic regression model, evaluating correlation with gender, age, degree of education, clinical condition and CD4+ T‐cell count at diagnosis and health centres, is shown in Table S3. Older age and being in follow‐up at TM were associated with a significant reduction of the risk to be early LTFU (*p* < 0.001 and *p* = 0.001 respectively), while male sex showed a positive correlation (*p* = 0.001).

When prescription approach (*immediate* vs. *early*, *where early is ART prescription within one month*) was added to the model (Table S4), a direct positive correlation between immediate ART prescription and early loss to follow‐up was at least suggested (AOR 1.7, 0.97–3.1, *p* = 0.064), where CSM remained another independent risk factor for early loss (AOR 2.7, *p* = 0.019).

When only women were considered in the analysis, pregnancy or breastfeeding preserved from early abandonment (AOR 0.44, *p* = 0.03), even if not when followed at CSM (AOR 3, *p* = 0.013) and in those with a low level of education (AOR 1.9, 0.99–3.6, *p* = 0.05).

### Retention in care

Older age displayed a protective role in retention in care after one year of follow‐up (AOR 1.24, IC95% 1.13–1.36, *p* < 0.001), while male sex and a low BMI were negatively correlated with retention in care (AOR 0.4, IC95% 0.24–0.65, *p* < 0.001 and AOR 0.25, IC95% 0.09–0.70, *p* = 0.01). Being followed in TM gave significant better results in compliance at the medical visit than MAH (AOR 4.65, IC95% 1.45–14.8, *p* = 0.009) (Table S5).

### ART adherence

Being pregnant or breastfeeding at the moment of HIV diagnosis showed a negative correlation, at least suggested, with adherence to treatment one year after prescription (AOR 0.37, IC95% 0.13–1.07, *p* = 0,068), and advanced WHO status (AOR 0.24, IC95%0.08–0.6, *p* = 0.005) (Table S6).

## DISCUSSION

Despite a good rate of ART prescription over one year of observation, HIV patients in rural Morrumbene in Mozambique display poor compliance to follow‐up and even worse adherence to antiviral treatment. In our study population, three of four patients were females. Besides, men were older than women, and tended to present with a more advanced stage of disease by the time of HIV diagnosis. Females are more represented than men in other African cohorts [11, 12, 13, 14, 15], and comprise 57% of the total HIV infections in the adult population of Mozambique [16]. Late HIV diagnosis in men has already been highlighted in other studies from Africa [12, 17, 18] and may be partly due to the different behaviour displayed by men in seeking help [19]. Consistent with another study from a similar cohort, more than 50% of patients presented with an advanced stage of disease (considered as a CD4+ T‐cell count <350 cells/ml), at diagnosis [12]. CSM accounted for the greatest proportion of patients diagnosed with an advanced clinical stage of disease (17%, *p* = 0.015), probably because this is the biggest health centre in the area and has an in‐patient ward for the most severe cases. Whether the fact that only 4–5% of patients in MAH and TM were clinically diagnosed with an advanced WHO status despite the high percentages of late presenters was due to a lack of recognition of WHO status III or IV defining diseases should be further investigated.

By the end of the observational period, 83% of all patients were prescribed ART. This is a much higher ART prescription rate than in other studies from Mozambique [2, 5, 20]. Consistent with national guidelines by the time of the study, pregnant women were more likely to be prescribed ART on the day of diagnosis (AOR 17, *p* < 0.001), whereas older patients and patients with higher T‐CD4+ cell counts tended to be prescribed ART later (AOR 0.2, 95%CI 0.12–0.33, *p* < 0.001 and AOR 0.86 for each 100, *p* < 0.001 respectively).

At 6 months, 49% of patients were present at the follow‐up visit, with 25% continuously adherent to ART. When we evaluated the proportion of patients who regularly attended all of their follow‐up visits after one year, we observed that only 34% were present at both visits and just 13% adhered to treatment.

These data are hardly comparable with other studies, mostly because the study design is highly heterogeneous among similar works in literature. In the SEARCH study [21], 88.6% were retained on care after one year, of which 78.5% had undetectable plasma levels of HIVRNA. In Tanzania, Uganda and Zambia, the proportion of patients retained in care after one year ranged from 52% to 96% [22]. In another cohort of patients diagnosed with HIV infection between 2007 and 2009 in Zimbabwe, the percentage of those who were still alive and prescribed with ART after one year was 78.1% [23]. However, in this case, adherence to treatment was not checked monthly. According to the IMASIDA estimates [2], in Mozambique, among those living with HIV infection currently on ART aging 15–49 years old, 68% had suppressed levels of plasmatic viraemia in 2015. In comparison with national estimates, our centres in Morrumbene seem to have a much lower rate of adherence to ART, and therefore, a much lower “presumed” viral suppression.

Among centres, TM had the highest adherence at follow‐up at six months (59%) and MAH recorded the worst ART adherence in the overall period. Factors influencing these results may be multiple, such as distance from the health centre, relationship between health practitioners and patients, awareness of patients about HIV treatment and follow‐up, perception of stigma and many others. Although the presence of MMI supporting the mobile clinic strategy could justify a better data register and a “motivational input” for rural populations, it is to be noted that the TM population presents peculiar characteristics, such as lower education and socio‐geographical isolation, justifying a lower probability to perceive the importance of a chronic therapy. These positive and negative factors may somehow offset each other. Exploring these matters in the near future may help in understanding these differences, while each centre may become more aware of its current situation and work on its weaknesses in the next years.

Among the possible factors influencing the retention into the HIV continuum of care, older age was protective against loss to follow‐up. This result is consistent with other studies, which found the same association [2, 20, 21, 22, 24]. On the contrary, male sex was associated with being early LTFU and with scarce retention in care, which is consistent with results from several cohorts [21, 22, 23, 25, 26, 27]. Whether this may be associated with cultural factors, as previously explored for HIV diagnosis [19], or due to higher rates of mortality due to late presentation, should be addressed in future studies.

Starting ART on the day of diagnosis of HIV infection was independently associated with early loss to follow‐up. In the Rapid Initiation of Treatment (RapIT) trial in South Africa, 64% of patients in the rapid arm (initiation of ART ≤90 days) were virally suppressed vs. 51% receiving the standard of care [28]. In the ANRS 12249 TasP trial, there were no significant differences in viral suppression between those initiating ART irrespective of the CD4+ T‐cell count and those following the standard of care (85.2%, in the intervention arm and 84.9%, in the control arm), while retention in care was only slightly higher in the intervention than in the control arm (86.2% versus 82.5%) [29]. These heterogeneous findings show that early initiation of ART may not be the only determinant of future retention in care; other cultural, logistic and structural factors may be more important [30].

Being pregnant or breastfeeding at the moment of HIV diagnosis was protective against loss to follow‐up, but showed a negative correlation (*p* = 0.068) with adherence to ART after one year. This result is consistent with other studies showing lower rates of retention in care in women who started therapy during pregnancy and/or breastfeeding than in women who undertook ART for their own health [24, 25, 26, 27]. Many factors have been described to play a role for ART retention among pregnant women, such as declining motivation to stay on ART after delivery, lack of clinical symptoms from HIV, lack of time due to caring for the newborn, new financial constraints and lack of support from the families, post‐partum depression and fear of disclosing HIV sero‐status to the partner because of possibility of separation and divorce. Among women who returned, involvement of their sexual partners was a strong motivation for adherence to follow‐up [25, 28, 29, 30].

Severe illness has been reported to be a motivator for enrolment in care, as improvement in health has been described as a strong factor for the continuation of HIV care [5]. In our study, advanced disease was associated with scarce adherence to the antiretroviral treatment, similar to other studies [22, 23, 25]. Unfortunately, we did not explore the causes of the association. Whether this may be due to a higher mortality in this group, as previously observed [31, 32], should be further evaluated.

This study has several limitations. First of all, the absence of a control group and of a prospective design of the study did not allow to draw reliable comparisons among the three health centres in order to better understand the differences and the efficacy among the different strategies of HIV services delivery. Secondly, despite a good sample size, our population was not representative of the overall HIV‐positive population living in the district of Morrumbene, where we only described the population diagnosed in 2017 in three health centres. Another limitation of our study is the duration of follow‐up. As others already highlighted [5, 33, 34], HIV‐positive patients may engage, then drop out, then re‐engage in HIV services during their life. Therefore, a higher duration of follow‐up may give a more representative picture of the state of the retention in care among HIV‐positive patients. Moreover, as already stated, a considerable amount of data in medical records were missing. Considering the mortality rates and proportion of lost patients of other studies in similar contexts [5, 31, 32], the proportion of patients dead or referred to other centres seems underestimated in our study, due to underreporting, which may have increased the rates of LTFU.

Anyway, given all these limitations, we think that this study highlights important results: retention in care, and most of all, adherence to antiretroviral treatment need to be carefully addressed in this area. HIV‐positive men need special attention, particularly as regards early diagnosis and long‐term adherence to follow‐up. Newly HIV diagnosed mothers and their children should be carefully followed, especially after birth, where any factors associated to LTFU in this population should be understood and addressed.

## CONCLUSIONS

Despite ART being proposed to a high proportion of patients diagnosed with HIV infection, retention in care and adherence to ART after one year display meagre results. Given that early initiation of ART has already proven its benefits (44), this strategy should probably be complemented with other interventions. Men need to be especially addressed in future strategies to diagnose them at an earlier stage. Particular attention must be paid to younger patients, pregnant and breastfeeding women, especially long‐term, when the reasons of the losses to follow‐up in these populations should be understood and addressed. We hope that routine testing of HIV viral load will be soon available to help manage HIV‐positive patients in this area and to understand the efficacy of ART in this population.

## DECLARATIONS

The data supporting the findings of this study are available from the corresponding author upon reasonable request. PM received financial support from MMI for travel to and living in Mozambique during data collection. LRT and ST declare no conflicts of interest. FC reports acting as a principal investigator of company‐sponsored clinical trials in the field of HIV infection (ViiV Healthcare, GlaxoSmithKline, Gilead Sciences and Janssen – Cilag). CC and ADG were working as health providers for MMI by the time of the study. The study was conducted in the context of the health cooperation project "CAREvolution: Innovation of community health services in Inhambane Province" (AID Code 11492), in which MMI activities were financially supported by the Italian Agency for International Development Cooperation (AICS).

## Supporting information

Supplementary Material
