# Supplementary material for: The cascade of care of HIV after one year of follow‐up in a cohort of HIV‐positive adult patients in three health settings of Morrumbene in rural Mozambique
Source: Trop Med Int Health. 2021 Sep 12;26(11):1503–11. doi: 10.1111/tmi.13671 (PMC9293170; doi:10.1111/tmi.13671)
Supplement: Supplementary file 1 — Supplementary Material [file TMI-26-1503-s001.docx]

**Table 2**. Factors associated with loss to follow-up. Monovariate logistic regression.

| Factors | OR | IC95% | p |
| --- | --- | --- | --- |
| Male | 1.4 | 1.02-1.84 | 0.033 |
| Age (or every 5 years) | 0.86 | 0.82-0.91 | <0.001 |
| Number of cohabitants | 1.03 | 0.97-1.1 | 0.28 |
| Not schooled | 0.7 | 0.5-1 | 0.058 |
| Primary school | 1.1 | 0.8-1.5 | 0.45 |
| Secondary or higher school | 1.5 | 1.07-2 | 0.015 |
| Under-weight (BMI<18.5) | 1.8 | 1.03 -3.1 | 0.039 |
| WHO stage I | 0.8 | 0.6-1.09 | 0.17 |
| WHO stage II | 1 | 0.8-1.4 | 0.69 |
| WHO stage III or IV | 1.3 | 0.9-1.9 | 0.14 |
| CD4+ T-cells <200 cell/μl | 0.9 | 0.7-1.2 | 0.53 |
| For every 100 CD4+ T-cells | 1.02 | 0.97-1.07 | 0.35 |
| CD4+ T-cells <200cell/μl + WHO stage >II | 1.5 | 0.92-2.4 | 0.10 |
| Pregnancy or breastfeeding in first 6 months | 0.6 | 0.4-0.97 | 0.035 |
| cART prescription on the day of HIV diagnosis vs 7-30 days later | 2.2 | 1.4-3.3 | <0.001 |
| Early cART prescription | 0.5 | 0.4-0.7 | <0.001 |
| Follow-up at CSM | 1.5 | 1.1-2 | 0.007 |
| Follow-up at TM | 0.6 | 0.4-0.9 | 0.025 |
| Follow-up at MAH | 0.8 | 0.5-1.1 | 0.18 |

**Table 3**. Stepwise logistic regression model for probability of early loss to follow-up (AUC ROC 0.66) 548 observations, Prob>Chi2<0.001 and a Log likelihood = -341.

| Factors | OR | IC95% | p |
| --- | --- | --- | --- |
| TM | 0.4 | 0.2-0.8 | 0.001 |
| Male | 1.9 | 1.3-2.9 | 0.001 |
| Age (for every 5 years) | 0.8 | 0.75-0.88 | <0.001 |
| Under-weight (BMI<18.5) | 1.8 | 0.9-3.5 | 0.07 |

**Table 4**. Stepwise logistic regression model for probability of loss to follow-up at 6 months (AUC ROC 0.69) 366 observations, Prob>Chi2<0.001 and a Log likelihood = -213.

| Factors | OR | IC95% | p |
| --- | --- | --- | --- |
| CSM | 2.7 | 1.2-6 | 0.019 |
| Male | 1.7 | 1.03-3 | 0.036 |
| Age (for every 5 years) | 0.8 | 0.71-0.88 | <0.001 |
| WHO II | 1.7 | 0.93-3 | 0.086 |
| CART *immediate* vs *early prescription* | 1.7 | 0.97-3.1 | 0.064 |

Table 5. Stepwise logistic regression model for probability of being on FU at 6 and 12 months (AUC ROC 0.67) 423 observations, Prob>Chi2<0.001 and a Log likelihood = -263.

| Factors | OR | IC95% | p |
| --- | --- | --- | --- |
| TM vs CSM | 1.03 | 0.34-2.0 | 0.9 |
| TM vs MAH | 4.65 | 1.45-14.8 | 0.009 |
| Male | 0.4 | 0.24-0.65 | <0.001 |
| Age (for every 5 years) | 1.24 | 1.13-1.36 | <0.001 |
| BMI <17 | 0.25 | 0.09-0.70 | 0.010 |

**Table 6**. Stepwise logistic regression model for probability of being adherent to treatment for 12 months (AUC ROC 0.66) 471 observations, Prob>Chi2<0.001 and a Log likelihood = -172.

| Factors | OR | IC95% | p |
| --- | --- | --- | --- |
| CSM vs TM | 1.6 | 0.53-4.8 | 0.41 |
| MAH vs TM | 0.38 | 0.06-2.24 | 0.29 |
| WHO stage >2 | 0.22 | 0.08-0.6 | 0.005 |
| Pregnant/breastfeeding | 0.37 | 0.13-1.07 | 0.068 |
| Second. or higher school | 0.6 | 0.32-1.13 | 0.11 |
